# Natural Products Targeting ER Stress, and the Functional Link to Mitochondria

**DOI:** 10.3390/ijms21061905

**Published:** 2020-03-11

**Authors:** Stefania Martucciello, Milena Masullo, Antonietta Cerulli, Sonia Piacente

**Affiliations:** 1 Dipartimento di Chimica e Biologia, Università degli Studi di Salerno, Via Giovanni Paolo II 132, 84084 Fisciano (Salerno), Italy; smartucciello@unisa.it; 2 Dipartimento di Farmacia, Università degli Studi di Salerno, Via Giovanni Paolo II 132, 84084 Fisciano (Salerno), Italy; mmasullo@unisa.it (M.M.); acerulli@unisa.it (A.C.)

**Keywords:** ER stress, mitochondria, natural products, apoptosis, unfolded protein response, Ca^2+^ permeability transition

## Abstract

The endoplasmic reticulum (ER) is a dynamic organelle essential for intracellular homeostasis maintenance, controlling synthesis, the folding of secreted and membrane-bound proteins, and transport of Ca^2+^. During cellular stress, ER dysfunction leads to the activation of unfolded protein response (UPR) due to accumulated misfolded proteins in the ER. This condition is referred as ER stress. Mitochondria and ER form a site of close contact (the mitochondria-associated membrane, MAM) which is a major platform exerting important physiological roles in the regulation of intracellular Ca^2+^ homeostasis, lipid metabolism, mitochondrial fission, autophagosome formation, and apoptosis progression. Natural products have been receiving increasing attention for their ability to interfere with ER stress. Research works have focused on the capacity of these bioactive compounds to induce apoptosis by activating ER stress through the ER stress-mediated mitochondrial apoptotic pathway. In this review we discuss the role of natural products in the signaling communication between ER and mitochondria, focusing on the effects induced by ER stress including Ca^2+^ permeability transition and UPR signaling (protein kinase R-like ER kinase/mitofusin 2).

## 1. Introduction

Nature has been a source of bioactive compounds for millennia, with many useful drugs developed from plant sources. The continuing research of natural products with biological activity is due to their exceptional complexity and molecular diversity.

Over recent years an increasing number of plant metabolites have been investigated for their ability to affect endoplasmic reticulum (ER) stress, which has been documented to be linked to different diseases including inflammatory diseases, cancer, lung diseases, neurological diseases, diabetes and diabetic-related diseases/complications, malaria, and viral infections [1]. 

The ER is an important organelle present in eukaryotic cells, and is involved in the maintenance of intracellular homeostasis processes including the synthesis and folding of secreted and membrane-bound proteins. In the ER lumen, chaperones and enzymes assist the folding of proteins to be translocated to the Golgi apparatus in “proper” folding; misfolded proteins are targeted for degradation [2,3]. The ER is also a suitable environment for lipids and steroids including cholesterol biosynthesis.

The ER is the central site for Ca^2+^ storage, considering that homeostasis and the folding protein reaction are related to the intralumenal Ca^2+^ level [4]. Several endogenous and exogenous factors may determine the alteration of cellular conditions, inducing variations in ER homeostasis and its appropriate functioning. This can cause a cascade of signaling events activating an adaptive pathway to re-establish ER homeostasis, inducing an unfolded protein response (UPR) to mitigate the ER stress condition. Activating transcription factor 6 (ATF6), protein kinase R-like ER kinase (PERK), and inositol-requiring enzyme 1 (IRE1) are the three main transmembrane proteins initiating UPR signaling in ER stress response [5]. In normal conditions they are maintained in an inactive state. When ER stress is triggered, they are activated through dissociation from GRP78/binding protein (BIP), the master regulator chaperone of UPR, which aids proper folding of nascent polypeptides.

In particular, ATF6 is transported to the Golgi and in its cleaved form is translocated into the nucleus, inducing expression of transcription factors regulating overexpression of UPR elements such as ER-associated protein degradation (ERAD) to decrease unfolded protein accumulation and ER chaperones to stabilize protein folding [6,7]. 

PERK in its active form phosphorylates the α subunit of eukaryotic initiation factor 2 (eIF2α) which is related to protein translation of ATF4, inducing protein folding factor gene expression to regulate ER homeostasis. 

IRE1 is the most conserved modulator of the ER stress signaling pathway and exists in two isoforms, IRE1α and IRE1β, showing marked differences in tissue expression. IRE1α is expressed in all cell types and under stress conditions; in an active form it catalyzes the splicing of X-box-binding protein1 (XBP-1) in mature XBP1, involved in the regulation of protein folding. In contrast, IRE1β expression has been reported only in the gastrointestinal epithelium [8]. However, if the UPR is unable to restore homeostasis, ER stress induces apoptotic pathways [9]. 

**Figure 1:**
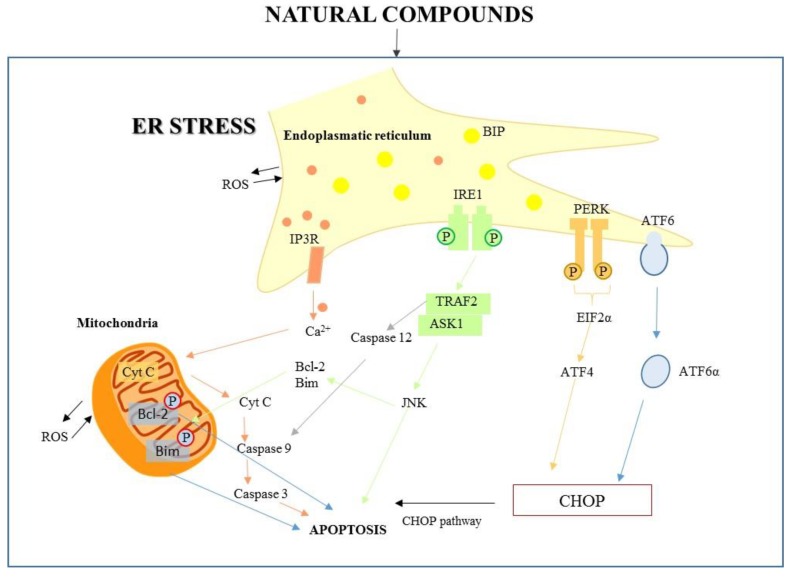
The endoplasmic reticulum (ER) signaling pathway and the ER–mitochondria pathway triggered by natural compounds.

The first key molecule for ER stress-mediated apoptosis is the C/EBP homologous protein (CHOP). PERK activation induces eIF2 phosphorylation, which activates ATF4, which subsequently determines the upregulation of proapoptotic factor CHOP; its expression is increased also by ATF6. Growth arrest, DNA-damage-inducible protein 34 (GADD34), and endoplasmic reticulum oxireductin 1 (ERO1α) are transcriptional targets of CHOP; they increase cellular reactive oxygen species (ROS) production and Ca^2+^ concentration. Therefore, CHOP decreases the antiapoptotic expression of B-cell lymphoma 2 (Bcl-2) and B-cell lymphoma extra-large (Bcl-xl), and increases expression of Bcl-2 homologous antagonist/killer (Bak), Bcl-2-associated X protein (Bax), phorbol-12-myristate-13-acetate-induced protein 1 (Noxa), Bcl-2-like protein 11 (BIM), and p53 upregulated modulator of apoptosis (PUMA) [10,11]. The ER stress-apoptotic pathway can activate IRE1 and the subsequent signaling pathway, considered one of the main UPR pathways. In the pro-apoptotic branch, IRE1 binds tumor necrosis factor (TNF) receptor-associated factor 2 (TRAF2), and the IRE1α-TRAF2 complex triggers caspase-12, activating in turn the apoptosis effector caspase-3. IRE1α-TRAF2 activates apoptosis signal-regulating kinase 1 (ASK1), inducing phosphorylation of c-jun-N terminal kinase (JNK) to activate the pro-apoptotic protein cJUN NH2-terminal kinase. JNK plays a role in apoptosis by stimulating pro-apoptotic (Bak and Bax) proteins and inactivating anti-apoptotic Bcl-2 protein [12]. These pathways are illustrated in Figure 1.

Therefore, ER stress-induced apoptosis could be an important strategy for many therapeutic approaches. 

Increasing evidence suggests that natural products target the ER stress signaling pathway, exerting a possible role in the prevention of cancer, metabolic diseases, cardiovascular diseases, and neurodegenerative diseases like Alzheimer’s and Parkinson’s [13,14,15]. Natural compounds are able to exert their effects both by inducing chronic ER stress or reducing ER stress; thus, on the basis of their mechanism of action they can be considered for the treatment of the above-mentioned pathologies. In particular, several studies showed that natural compounds targeting components of UPR and reducing ER stress play an important role in heart failure, ischemic heart disease, and atherosclerosis, representing a potential approach to treat cardiovascular diseases [15]. Recent studies on neurodegenerative diseases show that ER stress, in conjunction with abnormal protein degradation, can contribute to the pathophysiology of Parkinson’s disease, while the involvement of the ER due to the occurrence of amyloid β-peptide has been hypothesized in Alzheimer’s disease, taking into account that this peptide is synthesized and accumulates in the ER [16]. Natural products acting as ER modulators, either ER stress inducers or ER protectors, have been shown to exert a protective effect on neurodegenerative diseases [16]. 

Besides these reports, the role of natural products targeting ER stress in the cancer is increasingly being investigated. These studies also demonstrate that natural products can exert anticancer activity, by inducing chronic ER stress, or by inhibiting ER stress-related proteins to reduce the adaptative UPR. 

Considering the great attention being placed on this topic, here an update of recent works on natural products and their ability to interfere with ER stress is reported, with particular attention on compounds able to target ER stress and its functional link to mitochondria.

## 2. ER–Mitochondria Interactions 

The ER is an organelle sensitive to stress conditions. It is able to rapidly activate molecular mechanisms to restore cellular homeostasis and induce apoptotic signals. Mitochondria are dynamic organelles controlling a variety of biological events including energy production and regulation of apoptosis. Their number and morphology can be remodeled by fusion and fission events in response changing environmental conditions [17]. The ER and mitochondria are dynamic organelles working within a highly integrated reticular network involved in a variety of biological events [17]. Data indicate that ER and mitochondria cooperate in apoptosis signaling through sites of close contact called mitochondria-associated ER membranes (MAMs), acting as a platform of various signaling pathways crucial for the regulation of cellular homeostasis [15,18,19]. 

### 2.1. ER–Mitochondrial Interaction by Ca^2+^ Signaling Dependent Mechanisms

The most important effect of ER and mitochondria interaction is on modulation of Ca^2+^ dependent mechanisms, which play an important role in maintaining control of survival/death pathways. ER stress-induced Ca^2+^ release into cytosol causes depolarization of the inner mitochondrial membrane, leading to mitochondrial reactive oxygen species (ROS) formation which in turn facilitates accumulation of misfolded proteins within ER by impairing protein processing, which may promote ER stress and may lead to dilation of ER. In addition, the subsequent mitochondrial Ca^2+^ influx is considered the leading cause of mitochondrial swelling [20].

Under normal physiological conditions, both cytosolic Ca^2+^ and Ca^2+^ stored within the ER lumen are tightly regulated. Import of Ca^2+^ into ER is maintained by sarcoplasmic/ER Ca^2+^ ATPase (SERCA) pumps. During cellular stress events requiring a Ca^2+^-signal, Ca^2+^ is released from ER stores via ryanodine receptors (RyR) and in particular by the IP3Rs that have recently been reported as primarily clustered in the MAM regions where the ER is closely juxtaposed to mitochondria, delineating these zones as primary subcellular microdomains of Ca^2+^-transfer from the ER to mitochondria [21]. Ca^2+^ uptake into mitochondria is controlled by specific proteins such as the voltage-dependent anion channel (VDAC), resident on the outer mitochondrial membrane (OMM), mediating Ca^2+^ transfer between the two organelles. Additionally, mitochondria and associated proteins including the mitochondrial Ca^2+^ uniporter (MCU) and the mitochondrial Na^+^/Ca^2+^ exchanger (mNCX) are also related to mitochondrial Ca^2+^ regulation. Sustained high levels of Ca^2+^ in mitochondria cause the release of cytochrome c apoptotic protease activating factor-1 (Apaf-1), and ATP binds to procaspase 9, leading to apoptosome formation and activation of caspase 9, which in turn activates caspase 3, triggering death signals [22].

### 2.2. Cross-Talk UPR-Signaling to Mitochondria

The functional relationship between ER and mitochondria is essential to maintaining cellular homeostasis. ER is an activator of apoptosis signaling when elevated accumulation of unfolded proteins induces prolonged ER stress response. In this condition the ER promotes apoptosis through a mitochondrial pathway. In particular, as highlighted by Beukes et al., the activation of IRE1 as mediator of the UPR signaling pathway can induce apoptosis either via the phosphorylation of Bcl-2 and Bim, or via activation of the mitochondria-dependent pathway leading to cytochrome c release, apoptosome formation, and caspase activation. Data suggest that mitochondrial dysfunction increases ER stress levels, inducing up-regulation of JNK signaling activation [23].

PERK, an UPR-related protein, has been shown to reside in MAM where it exerts functions independent of its kinase activity [24]. In MAMs, PERK interacts with mitofusin 2 (MFN2), a GTPase protein involved in the mitochondrial fusion process. The PERK-MFN2 connection has an important structural function in increasing the contact surface between the ER and mitochondria. Moreover, PERK is involved in mitochondrial ROS-mediated apoptosis through MFN2 interaction [25]. 

## 3. Natural Products and the Signaling Communication between ER and Mitochondria

**Figure 2:**
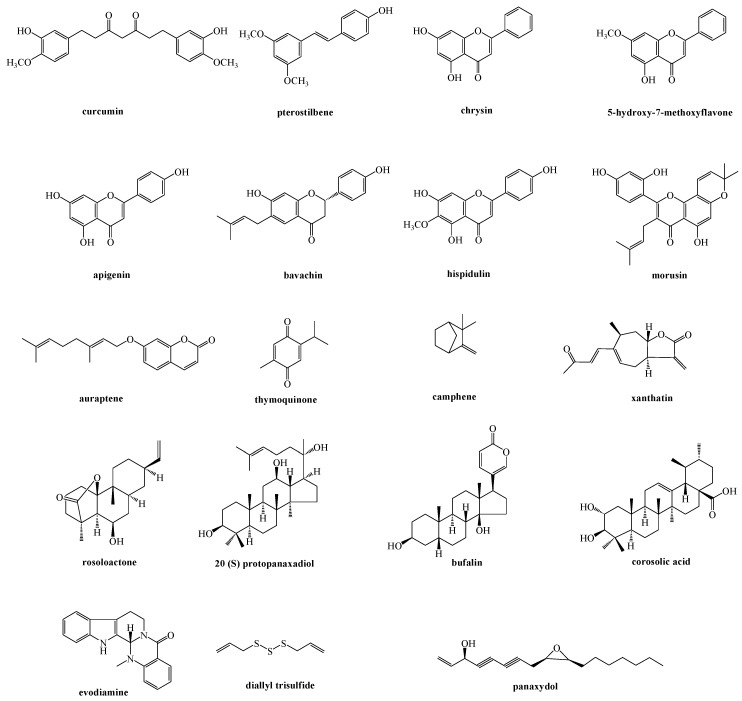
Natural compounds acting on the signaling communication between the ER and mitochondria.

**Figure 3:**
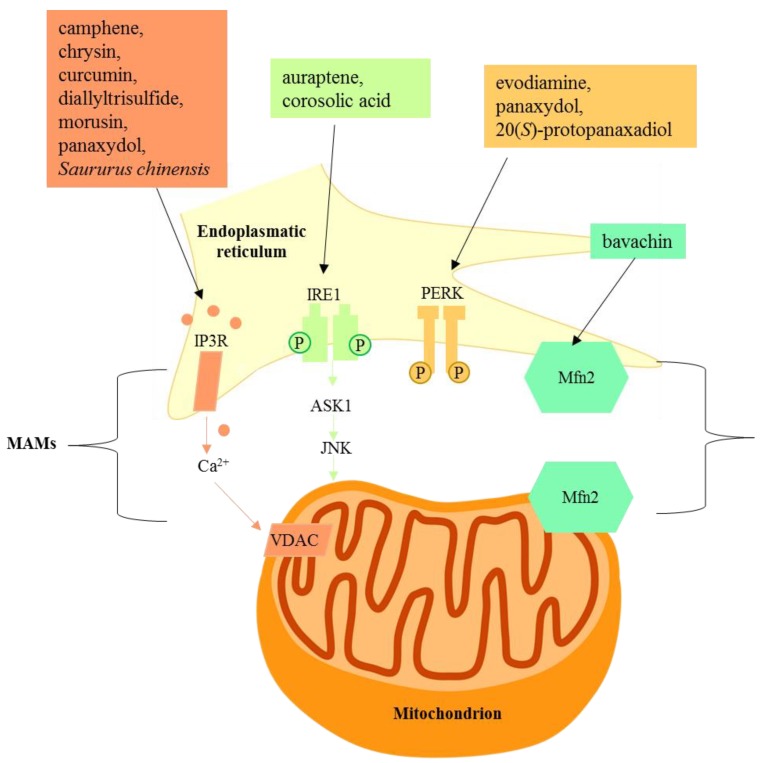
Natural products targeting mitochondria-associated membranes.

Different natural compounds have been reported to induce ER stress-related apoptosis by the mitochondria pathway, and among them flavonoid derivatives represent the main investigated class. Other natural products able to act on the signaling communication between the ER and mitochondria, classified on the basis of their chemical features, have been reported (Figure 2 and Figure 3).

### 3.1. Flavonoid Derivatives and Phenolic Compounds

Flavonoids are polyphenolic compounds ubiquitous in nature. More than 4000 flavonoids have been recognized, many of which are found in vegetables, fruits, and beverages like tea, coffee, and fruit drinks. Flavonoids have gained much attention because of their broad biological and pharmacological activities including antimicrobial, cytotoxic, anti-inflammatory, and cancer-preventing activities [26,27,28,29]. 

Morusin is a flavonoid reported in the root bark of *Morus australis* (Moraceae), characterized by two prenyl functions at positions 3 and 8. Previous investigations highlighted its anti-inflammatory, antioxidant, and antibacterial activities, along with antiproliferative activity against several human cancer cell lines. Recently, an investigation carried out on human epithelial ovarian cancer treated with morusin showed that morusin was able to induce ER stress by increasing the expression of binding immunoglobulin protein (BiP, a heavy-chain-binding protein), CHOP, IRE1α, and phosphorylated eukaryotic initiation factor alpha subunit (p-eIF2α). Moreover, cells resulted in a paraptosis-like cell death, characterized by the dilation of the ER and mitochondria, due to the release of Ca^2+^ from the ER to mitochondria [30]. 

Bavachin is a natural product belonging to the flavonoid class, isolated from *Psoralea corylifolia* (Fabaceae). In a recent investigation, bavachin significantly inhibited cell proliferation in human hepatocellular carcinoma (HepG2) cells, inducing apoptosis mainly through ER stress and mitochondrial apoptosis. The role of Mfn2 in bavachin-induced ER stress and UPR signaling was explored. The results showed how the HepG2 cells exhibited a gradual decrease in the Mfn2 protein levels and how small interfering RNA (siRNA) knock-down of Mfn2 resulted in a remarkable aggravation of ER stress through the inhibition of protein kinase B (Akt) phosphorylation. Along with these observations, results also displayed how the suppression of ROS by ROS scavengers reduced bavachin-induced ER stress [31].

5-hydroxy-7-methoxyflavone, a chrysin derivative isolated from *Kaemperia parviflora* (Zingiberaceae), was reported to induce cytotoxicity in the human colon cancer cell line HCT-116. Chemically, the hydroxyl group at 5-position of the flavonoid skeleton plays a pivotal role in the inhibition of cancer cell proliferation. The chemical features of the compound make possible its binding to the cellular plasma membrane, resulting in enhanced uptake into the cytosol. In detail, the treatment of HCT-116 cell line with 5-hydroxy-7-methoxyflavone led to ROS generation and Ca^2+^ release, resulting in ER stress induction. Simultaneously, 5-hydroxy-7-methoxyflavone causes alterations in mitochondrial membrane potential (MMP) and a reduction of the Bcl-2/Bax ratio, leading to activation of caspase-3 and apoptosis progression [32]. 

Auraptene, a prenylate coumarin isolated from the leaves of the aromatic plant *Zanthoxylum schinifolium* (Rutaceae), showed an apoptotic effect against the human acute leukemia Jurkat T cell line. This apoptotic effect was exerted by ER stress-mediated activation of caspase-8, caspase-12, and JNK. Among these effects, caspase 8 activation seems to be relevant for the subsequent caspase cascade activation. In fact caspase-8, along with JNK activation, determines mitochondrial cytochrome c release. In this way the involvement of mitochondria in the apoptotic effect caused by auraptene on the human acute leukemia Jurkat T cell line was explained [33].

Along with flavonoid derivatives, curcumin, a natural polyphenolic compound isolated from rhizome of *Curcuma longa* (Zingiberaceae) [34], has been extensively investigated for its role in activation of ER stress-related apoptosis in cancer cells [13]. Curcumin and its analogues have been found to exhibit therapeutic efficacy in patients with several types of progressive advanced cancers. In a recent work, after 24-h exposition of human papillary thyroid carcinoma BCPAP cells to curcumin, an increase of intracellular Ca^2+^ content in cells was observed due to inhibition of the sarco-endoplasmic reticulum ATPase 2A (SERCA2) pump. The increased cytosolic Ca^2+^ then linked calmodulin to activate calcium/calmodulin-dependent protein kinase II (CaMKII) signaling, leading to mitochondrial apoptosis pathway activation. So, curcumin induces apoptotic cell death by increasing ER stress and mitochondrial dysfunction [20]. 

Based on the activity of curcumin, a library of 23 achiral curcumin analogs was tested on human non-small-cell lung carcinoma (A549), hepatocellular carcinoma (HepG2), and pancreatic cancer (PANC-1) cell lines. These compounds were able to cause ER stress, leading to the induction of the unfolded protein response, of mitochondrial membrane depolarization, of caspase-3 activation, and subsequently of DNA breakdown in pancreatic cancer cells [35].

### 3.2. Terpene Derivatives

Terpenoids represent the largest groups of plant metabolites, mainly occurring in plants as constituents of essential oils. Despite their enormous diversity in structures and functions, all terpenoids derive from the same basic five-carbon unit, formally deriving from the hydrocarbon isopentenyl pyrophosphate (IPP) and its isomer dimethylallyl pyrophosphate (DMAPP). They are classified according to the number of isoprene units as monoterpenes (C10), sesquiterpenes (C15), diterpenes (C20), sesterterpenes (C25), triterpenes (C30), tetraterpenes (C40), and polyterpenes [26,36]. 

Camphene, a natural monoterpene isolated from the essential oil of *Piper cernuum* (Piperaceae) leaves, showed cytotoxicity in human cancer cell lines and in B16F10-Nex2 murine melanoma cells by in vitro and in vivo assays. The relation between camphene and the induction of intrinsic apoptosis in melanoma cells by ER stress with the release of Ca^2+^, chromatin, and chaperone proteins, as well as loss of mitochondrial membrane potential and up-regulation of caspase-3 activity, have been reported [37]. 

Rosoloactone, a diterpenoid isolated from the endophytic fungus *Trichothecium roseum*, showed marked anticancer activity against human cervical cancer HeLa cells. This effect was associated to ER stress and mitochondrial damage. The treatment of HeLa cells with rosoloactone caused an increase of ROS levels which is reported to play an important role in apoptosis induction. Moreover, the apoptotic effect exerted by rosoloactone could be associated to its ability to exert ER stress, inducing the accumulation of misfolded proteins [38]. 

Corosolic acid is a pentacyclic triterpene, isolated from the leaves of *Eryobotria japonica* (Rosaceae), from the fruit of *Cratoegus pinnatifida* var. psilosa (Rosaceae), and from the roots of *Actinidia chinensis* (Actinidiaceae). Corosolic acid was reported to suppress cell proliferation and to induce apoptosis in human prostate cancer cell lines PC-3 and DU145. In detail, the treatment of cancer cell lines with corosolic acid caused ER stress by two pro-apoptotic signaling pathways, namely IRE-1/ASK1/JNK and PERK/eIF2α/ATF4/CHOP. IRE-1 possesses kinase activity by the IRE-1-TRAF2-ASK1-JNK pathway, which induces mitochondrion-dependent intrinsic apoptotic pathway. In particular, IRE1 binds TRAF2, determining ASK1 phosphorylation and JNK phosphorylation, causing the activation of mitochondrion-dependent intrinsic apoptotic pathway. Moreover, corosolic acid activated the PERK-eIF2a-ATF4 pathway, leading to the pro-apoptotic CHOP up-regulation by inhibition of anti-apoptotic kinase AKT [39]. 

20(*S*)-Protopanaxadiol, a triterpene aglycone of the glycosides occurring in *Panax ginseng* (Araliaceae) known as ginsenosides, inhibited cell growth and induced apoptosis in human hepatocarcinoma HepG2 cells. After treatment with 20(*S*)-protopanaxadiol, the cells showed a massive cytoplasmic vacuolization and a dramatic change of ER morphology. The induction of ER stress was associated with the upregulation of ER stress-associated genes and proteins. 20(*S*)-Protopanaxadiol activated UPR through the phosphorylation of PERK and eIF2α, the splicing of XBP1 mRNA, and the cleavage of AFT6. Moreover, 20(*S*)-protopanaxadiol has been demonstrated to induce the intrinsic and extrinsic apoptotic pathways through the activation of DR5, and caspase-8, -9, -3, and to support the cleavage of PARP, the downregulation of Bcl-2, Bcl-xL, and MMP [40]. 

### 3.3. Miscellaneous

Evodiamine is a quinolone alkaloid isolated from the traditional herbal medicine *Euodia rutaecarpa* (Rutaceae). Evodiamine inhibits the viability of human ovarian cancer cells A2780, A2780CP, ES-2, and SKOV-3 via activation of ER stress proteins such as JNK and PERK. Increased phosphorylation of JNK and PERK proteins by evodiamine was observed in ovarian cancer cells, and application of JNK inhibitors, including SP600125 (SP), jnk inhibitor (JNKI), and PERK inhibitor GSK260641, significantly inhibited evodiamine-induced apoptosis in human ovarian cancer cells, confirming the role of JNK, PERK, and caspase 3 in the cytotoxic activity of evodiamine. Moreover, Chen et al. reported as JNK and PERK activation by evodiamine disrupts mitochondrial membrane potential leading to apoptosis of human ovarian cancer cells [41]. 

Panaxydol, a C17 polyacetylenic compound isolated from *Panax ginseng* (Araliaceae) roots, induced apoptosis in cancer cell lines and inhibited in vivo tumor growth in syngeneic and xenogeneic mouse tumor models; moreover, it displayed an in vivo anticancer effect against the mouse cancer cell line Renca (renal carcinoma) and human cancer cell line PC3 (prostate cancer). The level of Ca^2+^ significantly increased after exposure to panaxydol, leading to oxidative stress and to subsequent ER stress for mitochondrial [Ca^2+^] uptake. 

Panaxydol caused ER stress-induced mitochondrial apoptosis. The reported data highlighted how the treatment with panaxydol caused apoptosis by the involvement of PERK, but not IRE1α or ATF6. The activation of PERK showed a dual role in the delivery of apoptotic signals from ER to mitochondria. In particular, it induced CHOP factor through PERK-eIF2a-ATF4 signaling, and it activated PERK localized in the MAM in a kinase activity-independent manner [42]. 

Diallyltrisulfide, an organosulfur compound of garlic oil, was reported to cause apoptosis of basal cell carcinoma (BCC) due to induction of ER stress and mitochondrial dysfunction. In detail, diallyltrisulfide induced cytosolic Ca^2+^ mobilization and ER stress-related molecules such as Bip/GRP78 and CHOP/GADD153, leading to caspase-4 and caspase-9 activation before causing apoptosis. 

Western analysis showed how the treatment of BCC cells with diallyltrisulfide increased the expression of the apoptotic factors phospho-p53 and proapoptotic Bax and at same time decreased the expression of the antiapoptotic molecules Bcl-2 and Bcl-xl in the examined cells. Diallyltrisulfide provoked the release of cytochrome c, an apoptosis-inducing factor, and HtrA2/Omi into the cytoplasm; moreover, it caused the activation of the caspase cascade and of some factors involved in the apoptosis but not dependent on caspase. Thus, the mechanism of action of diallyltrisulfide involved nuclear translocation of apoptotic-inducing factor, endonuclease G, and caspase-dependent factors [43].

### 3.4. Herbal Extracts 

Herbal extracts are combinations of several compounds which are able to contribute to their overall therapeutic effect. The presence of more substances contained in the phytocomplex can determine a synergistic action and may reduce the risk of toxicity, producing more complete and less drastic pharmacological effects than those of one or a few of its pure components. 

Lee et al. reported ER stress-mediated apoptosis induced by *Saururus chinensis* (Saururaceae), a perennial herbal plant cultivated in China and Korea. This herbal extract induced apoptosis of hepatocellular carcinoma HepG2 cells via oxidative stress. *Saururus chinensis* also caused CHOP activation by dissociating the binding immunoglobulin protein (BiP) from IRE1α and inducing Bax. Moreover, *Saururus chinensis* caused ER Ca^2+^ leakage into the cytosol; this last effect is known to play an important role in activating apoptosis. In fact, the influx of Ca^2+^ to the mitochondria activates the release of cytochrome c along with the mitochondrial pathway related to activation of downstream caspases, especially caspase-3. Furthermore, caspase-3 is necessary for PARP cleavage, an important marker of apoptosis [44].

Brazilian red propolis, elaborated by honey bees, is commonly used in folk medicine. Ethanol extracts of propolis were reported to induce apoptosis, mitochondrial dysfunction, ER stress, caspase-3 activity, and DNA fragmentation in human breast cancer cells (MCF-7) and in human fibroblasts. In treatment with Brazilian red propolis, daidzein and biochanin A, the main constituents of the ethanol extract of Brazilian red propolis, induced MCF-7 cell apoptosis through ER stress-related signaling due to induction of CHOP expression in MCF-7 cells and not in fibroblasts. Moreover, after treatment of MCF-7 cells with Brazilian red propolis, the expression of Bax mRNA in MCF-7 cells was increased, while Bcl-xL and Bcl-2 mRNA expression was decreased. In this way mitochondrial dysfunction was observed, since the increase of pro-apoptotic protein Bax as well as the decrease of anti-apoptotic proteins Bcl-xL and Bcl-2 are considered to be associated with the induction of apoptosis [45].

Grape seed proanthocyanidins, a group of flavonoids, have been shown to exert neuroprotective effects against oxygen-glucose deprivation/reoxygenation injury in mouse neuroblastoma N2a cells by different mechanisms. The anti-apoptotic effects of grape seed proanthocyanidins could involve a decrease in ER stress and mitochondrial dysfunction. 

In detail, the extract inhibited cell apoptosis of mouse neuroblastoma N2a cells. This effect can be explained with the decrease of the levels of CHOP, GRP78, and caspase-12, with the reinstatement of mitochondrial membrane potential and ATP generation in the cells treated with grape seed proanthocyanidins. In addition, the extract improved the activity of endogenous antioxidant factors and caused a decrease in ROS levels [46].

## 4. Natural Compounds Targeting ER Stress-Mediated Apoptosis: “An Update” 

In recent years several papers have focused on the role of ER stress in preventing cancer and on the potential anticancer properties of natural compounds able to induce ER stress-related death in cancer cells. In response to the stress state, cells have evolved an evolutionarily conserved signal transduction pathway (UPR), the primary aim of which is to restore ER homeostasis. However, when the stress conditions are too intense and cannot be restored, the UPR activates a cell death pathway. Several studies highlighted that natural products may exert their cytotoxic activity by inducing chronic ER stress as well as by inhibiting ER stress-related proteins to reduce adaptative UPR [13]. 

A recent review describes the ability of natural compounds like curcumin, resveratrol, green tea polyphenols, quercetin, garcinia xanthones and benzophenones, and tocotrienols, to exert ER stress-related anticancer activity [13]. Also, natural compounds belonging to terpenoids showed the ability to influence ER stress and homeostasis and to be involved in metabolic diseases influenced by ER. In detail, some terpenoids such as abscisic acid, lycopene, carotene, phytocannabinoids, geraniol, limonene, genipin, linalool, menthol, perillyl alcohol, kujigamberol, marrubiin, sarcodonin, ganoderiol F, artemisinin, dehydrocostus lactone, farnesol, parthenolide, and thapsigargin were reported to act with different proteins associated with ER stress, showing the ability to affect ER dysfunction and metabolic diseases such as diabetes, cancer, liver and neurological diseases, and parasitic infections [14]. 

Therefore, here we provide an update of natural compounds showing cytotoxic activity by ER stress-involving mechanisms. 

A phenolic compound recently reported for its ability to act on ER stress is apigenin, a flavonoid isolated from fruits and vegetables including chamomile, parsley, apples, and grapes. It is known for its potential therapeutic agent against diverse diseases such as diabetes, cardiovascular diseases, neuronal diseases, metabolic diseases, and cancers [47]. Apigenin was reported to induce apoptosis in two human endometriosis cell lines (VK2/E6E7 and End1/E6E7), in a dose-dependent manner, by disrupting mitochondrial membrane potential (MMP) which was accompanied by an increase of Ca^2+^ concentration in the cytosol. Moreover, apigen increased ROS levels, peroxidation of lipids in the cytosol, and levels of unfolded proteins. The treatment with apigenin in the two cell lines produced changes in proteins connected with ER stress, including PERK, eIF2α, IRE1α, GRP78, and GADD153. After treatment with apigenin, phosphorylation of above mentioned proteins increased in both endometriosis cell lines, while changes in the expression of IRE1α were not observed [47]. Hispidulin (4′,5,7-trihydroxy-6-methoxyflavone), a phenolic flavonoid isolated from *Salvia involucrate* (Lamiaceae), is reported to exert anti-cancer effects against a variety of solid tumors and hematological malignancies such as renal cell carcinoma, acute myeloid leukemia, gallbladder carcinoma, colorectal cancer, and hepatocellular carcinoma in in vivo and in vitro studies. With respect to hepatocellular carcinoma, it has been reported that hispidulin exerts anti-proliferative and pro-apoptotic effects in the hepatocellular carcinoma cell line HepG2. In particular, Han et al. reported that hispidulin dose-dependently inhibited cell growth and promoted the pro-apoptotic effect associated with mitochondrial pathway. In detail, hispidulin induced ER stress-mediated apoptosis in hepatocellular carcinoma cells by activating the 5′ AMP-activated protein kinase (AMPK)/mammalian target of rapamycin (mTOR) pathway. Treatment of cells with AMPK siRNA or an inhibitor significantly abrogated the effects of hispidulin on CHOP expression and apoptotic-related protein expression, providing evidence that hispidulin triggers ER stress (ERS) by modulating AMPK signaling [48].

Cirsimaritin is a flavonoid isolated from *Artemisia* spp. (Asteraceae), plants widely used in traditional medicine. It has been reported that cirsimaritin inhibited cell growth and induced mitochondrial apoptosis in gallbladder carcinoma (GBC-SD) cells. This effect depended on cirsimaritin-induced ROS production, which activates ER stress and mitochondrial apoptotic pathways. Moreover, cirsimaritin induced UPR activation firstly and then activated CHOP, which contributes to cell apoptosis by inhibition of anti-apoptotic kinase AKT. Inactivation of AKT may cause mitochondrial dysfunction by dephosphorylation of BCL2-associated agonist of cell death protein (Bad). ER stress and mitochondrial dysfunction induce activation of caspase-4 and -9, respectively and together cause cirsimaritin-induced GBC-SD cell apoptosis [49]. 

Xanthatin is a bicyclic sesquiterpene lactone isolated mainly from *Xanthium strumarium* (Asteraceae), and has shown significant anticancer activity in a variety of cell lines implicated in colon, breast, lung, cervix, and skin cancers. A recent investigation revealed that xanthatin arrested the cell cycle and induced apoptosis in human hepatoma cells. 

Xanthatin induced apoptosis in HepG2 cells by ER stress through activation of the UPR pathway. This effect was due to activation of the PERK/eIF-2α/ATF4 signaling pathway and to an increase of CHOP levels. Additionally, xanthatin induced apoptosis associated to activation of caspase-3. These effects were significantly abolished by siRNA-mediated knockdown of CHOP [50].

A monoterpene derivative, thymoquinone, is the main active compound of the volatile oil of *Nigella sativa* seeds (Ranunculaceae), which showed the ability to protect ischemia-reperfusion injury in liver through the attenuation of oxidative stress, mitochondrial damage, and ER stress and apoptosis, as well as an enhancement of P38 and ERK activation. These effects were determined by the reduction of the expression of ER stress parameters including GRP78, CHOP, and caspase-12, potentially linked to its antioxidant properties. Besides its effect on the ER stress, thymoquinone attenuated the expression of apoptotic parameters through the decrease of cytochrome C release and caspase-9 and caspase-3 activities [51].

Triptolide, a diterpene triepoxide isolated from *Tripterygium wilfordii* (Celastraceae), is reported to have different biological activities such as cytoskeleton dysfunction and disruption of cell–cell adherens junctions [52], inhibiting the expression of Rho GTPases such as RhoA and RhoB proteins involved in many processes like migration and angiogenesis [53]. Triptolide induced UPR by activating the PERK-EIF2α and IRE1-1αXBP pathways of UPR, leading to chronic ER stress in pancreatic cancer. The results showed that glucose-regulated protein 78 (GRP78) is downregulated by triptolide, causing cell death by apoptosis in MIA PaCa-2 cells. GRP78 is a master regulator of ER that has been reported to play important anti-apoptotic functions and that is frequently over-expressed at the surface of several cancer cells where it contributes to chemo-resistance [54]. Triptolide activates unfolded protein response leading to chronic ER stress in pancreatic cancer cells [55].

Cardiac glycosides are compounds with a steroidal nucleus possessing a lactone moiety at C-17 position, and a sugar chain at the C-3 position [56]. The nature of the lactone ring at C-17 defines the compound class, i.e., cardenolides (with an unsaturated butyrolactone ring) and bufadienolides (with an α-pyrone ring) [57,58]. Bufalin, a secondary metabolite belonging to cardiac glycoside class, exhibited cytotoxicity in U87MG and LN229 glioma cells, inducing mitochondrial-mediated apoptosis, autophagy, ER stress, and UPR. Shen et al. highlighted that mitochondrial- and ER mediated apoptosis were involved in glioma cell death caused by bufalin; moreover, bufalin caused autophagy by activating AMPK/mTOR and PERK/eIF2a/CHOP pathways. Therefore, this last effect reinforced the ER stress induced proapoptotic effect of bufalin [59].

Our recent data have demonstrated that 20-hydroxyecdysone and deglucohellebrin isolated from *Helleborus caucasicus* (Ranunculaceae) induced a down-regulation of GRP78 expression. For this reason, these compounds could represent good candidates in combination with other proapoptotic agents in anticancer therapies [60] 

Besides pure compounds, further studies were carried out to investigate the activity of plant extracts in ER stress. Trichosanthin, a traditional Chinese medicine obtained from the root tuber of *Trichosanthes kirilowii* (Cucurbitaceae), exhibited anticancer activities by inducing apoptosis in many different cancer cell lines. It has been demonstrated that the treatment of human leukemia cells (HL-60) with trichosanthin activates caspase 8, 9, and 3, involving both intrinsic and extrinsic apoptosis pathways. The involvement of mitochondria was demonstrated by reduction of mitochondrial membrane potential and the release of cytochrome c and Smac along with the activation of caspase-9. In addition, trichosanthin treatment induced upregulation of BiP and CHOP and also activated caspase-4, strongly supporting the involvement of the ERS pathway in trichosanthin-induced apoptosis. Therefore, trichosanthin induces human leukemia cell line apoptosis through both the caspase-9-dependent mitochondrial pathway and the caspase-4-dependent ER stress-triggered pathway. Both pathways directly activate caspase-3 or via caspase-8, which eventually leads to apoptosis in the human leukemia cell line [61]. 

## 5. Conclusions

The literature is providing increasing evidence on the involvement of ER stress in multiple pathogenic pathways (oxidative stress, inflammation, and apoptosis). Careful research has shown how in recent years there has been a growing number of manuscripts focused on the discovery of novel compounds able to improve multiple ER stress-mediated signaling pathways [15]. 

Mitochondria are organelles that produce ATP and many biosynthetic intermediates, also contributing to the metabolism of amino acids and lipids and to the maintenance of intracellular Ca^2+^ homeostasis. Approximately 5%–20% of the mitochondrial surface is in directly contact with the ER. Recent reports have identified a site of close contact between the ER and mitochondria, the MAM, characterized by several molecular bridges [18]. This site of direct communication between the ER and mitochondria is also defined as a subdomain of the ER. This region is important for lipid and Ca^2+^ trafficking between ER and mitochondria in order to regulate lipid metabolism and mitochondrial Ca^2+^ homeostasis. Recent studies highlight how in this region there are not only proteins correlated with lipid metabolism and Ca^2+^ trafficking but also MAM-resident proteins which make up a platform involved in several signaling pathways. Apoptotic, inflammatory, and antiviral innate immune responses as well as autophagic and metabolism-related signaling pathways are triggered by MAM-resident proteins. All these signaling pathways are closely related to both pro- and anti-oncogenic processes. So far, the exhaustive relationship between the MAM and cancer has not been defined and deeper investigations are required to extensively address this issue. 

The growing knowledge on the signaling between the ER and mitochondria has prompted the investigation of several natural compounds for their ability to interfere in this signaling. So far, compared with the investigation of natural compounds in ER stress, few manuscripts report how natural compounds are able to act on the specific signaling between ER and mitochondria. As is evident, the investigated compounds, belonging to different chemical classes, do not show an exclusive mechanism of action. Further studies are required to identify the chemical features responsible for the activity of these multiple signaling pathways, and research is expected to reveal new insights into novel natural compounds able to interfere with the mitochondria-associated ER membrane.

## Abbreviation

AMPK	5′ AMP-activated protein kinase
Apaf-1	Apoptotic protease activating factor-1
ATF4	Activating transcription factor 4
ATF6	Activating transcription factor 6
ASK1	Apoptosis signal-regulating kinase 1
Bak	Bcl-2 homologous antagonist/killer
Bax	Bcl-2-associated X protein
Bcl-2	B-cell lymphoma 2
Bcl-xl	B-cell lymphoma-extra large
BiP	Binding immunoglobulin protein
CHOP	C/EBP homologous protein
eIF2	Eukaryotic initiation factor 2
ER	Endoplasmic reticulum
ERAD	ER-associated degradation
ERO1	Endoplasmic reticulum oxireductin 1
GADD34	Growth arrest and DNA-damage-inducible protein 34
GRP78	Glucose-regulated protein 78
IRE1	Inositol requiring enzyme1
JNK	JUN N-terminal kinase
MAMs	Mitochondria-associated membranes
MCU	Mitochondrial calcium uniporter
MFN1	Mitofusin 1
MFN2	Mitofusin 2
MMP	Mitochondrial membrane potential
mNCX	Mitochondrial Na+/Ca2+ exchanger
mTOR	Mammalian target of rapamycin
OMM	Outer mitochondrial membrane
PERK	Protein kinase R-like ER kinase
PUMA	p53 upregulated modulator of apoptosis
ROS	Reactive oxygen species
RyR	Ryanodine receptors
SERCA	Sarcoplasmic/endoplasmic Ca^2+^ ATPase
TNF	Tumor necrosis factor
TRAF2	Tumor necrosis factor receptor-associated factor 2
UPR	Unfolded protein response
VDAC	Voltage-dependent anion channel
XBP-1	X-box-binding protein 1

## References

[1] Lin J.H., Walter P., Yen T.S. (2008). Endoplasmic reticulum stress in disease pathogenesis. Annu. Rev. Pathol..

[2] Ma Y., Hendershot L.M. (2004). The role of the unfolded protein response in tumour development: Friend or foe?. Nat. Rev. Cancer.

[3] Kincaid M.M., Cooper A.A. (2007). Misfolded proteins traffic from the endoplasmic reticulum (ER) due to ER export signals. Mol. Biol. Cell.

[4] Kim I., Xu W.J., Reed J.C. (2008). Cell death and endoplasmic reticulum stress: Disease relevance and therapeutic opportunities. Nat. Rev. Drug Discov..

[5] Amodio G., Moltedo O., Fasano D., Zerillo L., Oliveti M., Di Pietro P., Faraonio R., Barone P., Pellecchia M.T., De Rose A. (2019). PERK-Mediated Unfolded Protein Response Activation and Oxidative Stress in PARK20 Fibroblasts. Front. Neurosci-Switz..

[6] Gardner B.M., Walter P. (2011). Unfolded proteins are Ire1-activating ligands that directly induce the unfolded protein response. Science.

[7] Asada R., Kanemoto S., Kondo S., Saito A., Imaizumi K. (2011). The signalling from endoplasmic reticulum-resident bZIP transcription factors involved in diverse cellular physiology. J. Biochem..

[8] Martino M.B., Jones L., Brighton B., Ehre C., Abdulah L., Davis C.W., Ron D., O’Neal W.K., Ribeiro C.M.P. (2013). The ER stress transducer IRE1 beta is required for airway epithelial mucin production. Mucosal Immunol..

[9] Urra H., Dufey E., Lisbona F., Rojas-Rivera D., Hetz C. (2013). When ER stress reaches a dead end. Biochim. Biophys. Acta.

[10] Puthalakath H., O’Reilly L.A., Gunn P., Lee L., Kelly P.N., Huntington N.D., Hughes P.D., Michalak E.M., McKimm-Breschkin J., Motoyama N. (2007). ER stress triggers apoptosis by activating BH3-only protein Bim. Cell.

[11] Reimertz C., Kogel D., Rami A., Chittenden T., Prehn J.H. (2003). Gene expression during ER stress-induced apoptosis in neurons: Induction of the BH3-only protein Bbc3/PUMA and activation of the mitochondrial apoptosis pathway. J. Cell Biol..

[12] Malhi H., Kaufman R.J. (2011). Endoplasmic reticulum stress in liver disease. J. Hepatol..

[13] Limonta P., Moretti R.M., Marzagalli M., Fontana F., Raimondi M., Marelli M.M. (2019). Role of endoplasmic reticulum stress in the anticancer activity of natural compounds. Int. J. Mol. Sci..

[14] Beukes N., Levendal R.-A., Frost C.L. (2014). Selected terpenoids from medicinal plants modulate endoplasmic reticulum stress in metabolic disorders. J. Pharm. Pharmacol..

[15] Choy K.W., Murugan D., Mustafa M.R. (2018). Natural products targeting ER stress pathway for the treatment of cardiovascular diseases. Pharmacol. Res..

[16] Pereira D.M., Valentao P., Correia-da-Silva G., Teixeira N., Andrade P.B. (2015). Translating endoplasmic reticulum biology into the clinic: A role for ER-targeted natural products?. Nat. Prod. Rep..

[17] Lepretti M., Martucciello S., Aceves M.A.B., Putti R., Lionetti L. (2018). Omega-3 Fatty Acids and Insulin Resistance: Focus on the Regulation of Mitochondria and Endoplasmic Reticulum Stress. Nutrients.

[18] Kato H., Nishitoh H. (2015). Stress responses from the endoplasmic reticulum in cancer. Front. Oncol..

[19] Moltedo O., Remondelli P., Amodio G. (2019). The Mitochondria-Endoplasmic Reticulum Contacts and Their Critical Role in Aging and Age-Associated Diseases. Front. Cell Dev. Biol..

[20] Zhang L., Cheng X., Xu S., Bao J., Yu H. (2018). Curcumin induces endoplasmic reticulum stress-associated apoptosis in human papillary thyroid carcinoma BCPAP cells via disruption of intracellular calcium homeostasis. Medicine.

[21] Rizzuto R., Pinton P., Carrington W., Fay F.S., Fogarty K.E., Lifshitz L.M., Tuft R.A., Pozzan T. (1998). Close contacts with the endoplasmic reticulum as determinants of mitochondrial Ca^2+^ responses. Science.

[22] Varghese E., Samuel S.M., Sadiq Z., Kubatka P., Liskova A., Benacka J., Pazinka P., Kruzliak P., Busselberg D. (2019). Anti-Cancer Agents in Proliferation and Cell Death: The Calcium Connection. Int. J. Mol. Sci..

[23] Lee J.W., Kim W.H., Yeo J., Jung M.H. (2010). ER stress is implicated in mitochondrial dysfunction-induced apoptosis of pancreatic beta cells. Mol. Cells.

[24] Gilady S.Y., Bui M., Lynes E.M., Benson M.D., Watts R., Vance J.E., Simmen T. (2010). Ero1alpha requires oxidizing and normoxic conditions to localize to the Mitochondria-Associated Membrane (MAM). Cell Stress Chaperones.

[25] Munoz J.P., Ivanova S., Sanchez-Wandelmer J., Martinez-Cristobal P., Noguera E., Sancho A., Diaz-Ramos A., Hernandez-Alvarez M.I., Sebastian D., Mauvezin C. (2013). Mfn2 modulates the UPR and mitochondrial function via repression of PERK. EMBO J..

[26] Masullo M., Montoro P., Mari A., Pizza C., Piacente S. (2015). Medicinal plants in the treatment of women’s disorders: Analytical strategies to assure quality, safety and efficacy. J. Pharm. Biomed. Anal..

[27] Benavides A., Montoro P., Bassarello C., Piacente S., Pizza C. (2006). Catechin derivatives in *Jatropha macrantha* stems: Characterisation and LC/ESI/MS/MS quali-quantitative analysis. J. Pharm. Biomed..

[28] Cerulli A., Lauro G., Masullo M., Cantone V., Olas B., Kontek B., Nazzaro F., Bifulco G., Piacente S. (2017). Cyclic Diarylheptanoids from *Corylus avellana* Green Leafy Covers: Determination of Their Absolute Configurations and Evaluation of Their Antioxidant and Antimicrobial Activities. J. Nat. Prod..

[29] Masullo M., Mari A., Cerulli A., Bottone A., Kontek B., Olas B., Pizza C., Piacente S. (2016). Quali-quantitative analysis of the phenolic fraction of the flowers of *Corylus avellana*, source of the Italian PGI product “Nocciola di Giffoni”: Isolation of antioxidant diarylheptanoids. Phytochemistry.

[30] Xue J., Li R., Zhao X., Ma C., Lv X., Liu L., Liu P. (2018). Morusin induces paraptosis-like cell death through mitochondrial calcium overload and dysfunction in epithelial ovarian cancer. Chem. Biol. Interact..

[31] Yang Y., Tang X., Haa F., Ma Z., Wang Y., Wang L., Gao Y. (2018). Bavachin induces apoptosis through mitochondria! regulated ER stress pathway in HepG2 cells. Biol. Pharm. Bull..

[32] Ryu S., Lim W., Song G., Lim W., Bazer F.W. (2017). Chrysin induces death of prostate cancer cells by inducing ROS and ER stress. J. Cell. Physiol..

[33] Jun D.Y., Kim J.S., Park H.S., Han C.R., Fang Z., Woo M.H., Rhee I.K., Kim Y.H. (2007). Apoptogenic activity of auraptene of *Zanthoxylum schinifolium* toward human acute leukemia Jurkat T cells is associated with ER stress-mediated caspase-8 activation that stimulates mitochondria-dependent or -independent caspase cascade. Carcinogenesis.

[34] Iranshahi M., Chini M.G., Masullo M., Sahebkar A., Javidnia A., Yazdi M.C., Pergola C., Koeberle A., Werz O., Pizza C. (2015). Can Small Chemical Modifications of Natural Pan-inhibitors Modulate the Biological Selectivity? The Case of Curcumin Prenylated Derivatives Acting as HDAC or mPGES-1 Inhibitors. J. Nat. Prod..

[35] Szebeni G.J., Balazs A., Madarasz I., Pocz G., Ayaydin F., Kanizsai I., Fajka-Boja R., Alfoldi R., Hackler L., Puskas L.G. (2017). Achiral Mannich-Base Curcumin Analogs Induce Unfolded Protein Response and Mitochondrial Membrane Depolarization in PANC-1 Cells. Int. J. Mol. Sci..

[36] Masullo M., Pizza C., Piacente S. (2017). Oleanane derivatives for pharmaceutical use: A patent review (2000–2016). Expert Opin. Ther. Pat..

[37] Girola N., Figueiredo C.R., Farias C.F., Azevedo R.A., Ferreira A.K., Teixeira S.F., Capello T.M., Martins E.G.A., Matsuo A.L., Travassos L.R. (2015). Camphene isolated from essential oil of *Piper cernuum* (Piperaceae) induces intrinsic apoptosis in melanoma cells and displays antitumor activity in vivo. Biochem. Biophys. Res. Commun..

[38] Zhou L., Qin J., Ma L., Li H., Li L., Ning C., Gao W., Yu H., Han L. (2017). Rosoloactone: A natural diterpenoid inducing apoptosis in human cervical cancer cells through endoplasmic reticulum stress and mitochondrial damage. Biomed. Pharmacother..

[39] Ma B., Zhang H., Wang Y., Zhao A., Zhu Z., Bao X., Sun Y., Zhang Q., Zhang H., Li L. (2018). Corosolic acid, a natural triterpenoid, induces ER stress-dependent apoptosis in human castration resistant prostate cancer cells via activation of IRE-1/JNK, PERK/CHOP and TRIB3. J. Exp. Clin. Cancer Res..

[40] Zhu G.-Y., Li Y.-W., Tse A.K.-W., Hau D.K.-P., Leung C.-H., Yu Z.-L., Fong W.-F. (2011). 20(S)-Protopanaxadiol, a metabolite of ginsenosides, induced cell apoptosis through endoplasmic reticulum stress in human hepatocarcinoma HepG2 cells. Eur. J. Pharmacol..

[41] Chen T.-C., Chien C.-C., Wu M.-S., Chen Y.-C. (2016). Evodiamine from *Evodia rutaecarpa* induces apoptosis via activation of JNK and PERK in human ovarian cancer cells. Phytomedicine.

[42] Kim H.S., Lim J.M., Kim J.Y., Kim Y., Park S., Sohn J. (2016). Panaxydol, a component of *Panax ginseng*, induces apoptosis in cancer cells through EGFR activation and ER stress and inhibits tumor growth in mouse models. Int. J. Cancer.

[43] Wang H.-C., Hsieh S.-C., Yang J.-H., Lin S.-Y., Sheen L.-Y. (2012). Diallyl Trisulfide Induces Apoptosis of Human Basal Cell Carcinoma Cells via Endoplasmic Reticulum Stress and the Mitochondrial Pathway. Nutr. Cancer.

[44] Lee A.Y., Han Y.-A., Kim J.-E., Hong S.-H., Park E.-J., Cho M.-H. (2015). Saururus chinensis Baill induces apoptosis through endoplasmic reticulum stress in HepG2 hepatocellular carcinoma cells. Food Chem. Toxicol..

[45] Kamiya T., Nishihara H., Hara H., Adachi T. (2012). Ethanol extract of Brazilian red propolis induces apoptosis in human breast cancer MCF-7 cells through endoplasmic reticulum stress. J. Agric. Food Chem..

[46] Fu K., Chen L., Zhang W., Bai Y., Miao L., Guo Y. (2019). Grape Seed Proanthocyanidins Protect N2a Cells against Ischemic Injury via Endoplasmic Reticulum Stress and Mitochondrial-associated Pathways. CNS Neurol. Disord. Drug Targets.

[47] Park S., Song G., Park S., Song G., Lim W., Bazer F.W. (2018). Apigenin induces ROS-dependent apoptosis and ER stress in human endometriosis cells. J. Cell. Physiol..

[48] Han M., Gao H., Gao M.-Q., Liu K.-L., Xie J., Chen X.-H., Han Y.-T., Yuan Y.-P., Yuan Y.-P., Yuan Q. (2019). Hispidulin induces ER stress-mediated apoptosis in human hepatocellular carcinoma cells in vitro and in vivo by activating AMPK signaling pathway. Acta Pharmacol. Sin..

[49] Quan Z., Gu J., Dong P., Lu J., Wu X., Wu W., Fei X., Li S., Wang Y., Wang J. (2010). Reactive oxygen species-mediated endoplasmic reticulum stress and mitochondrial dysfunction contribute to cirsimaritin-induced apoptosis in human gallbladder carcinoma GBC-SD cells. Cancer Lett. (Shannon, Irel.).

[50] Shi T.-L., Zhang L., Feng X.-J., Cheng Q.-Y., Liu J., Shen Y.-J., Yu J.-S., Shen Y.-X. (2019). Xanthatin induces apoptosis by activating endoplasmic reticulum stress in hepatoma cells. Eur. J. Pharmacol..

[51] Bouhlel A., Mosbah B., Abdallah H., Ribault C., Viel R., Mannai S., Corlu A., Abdennebi B. (2017). Thymoquinone prevents endoplasmic reticulum stress and mitochondria-induced apoptosis in a rat model of partial hepatic warm ischemia reperfusion. Biomed. Pharmacother..

[52] Wang X., Zhao F., Lv Z.M., Shi W.Q., Zhang L.Y., Yan M. (2016). Triptolide disrupts the actin-based Sertoli-germ cells adherens junctions by inhibiting Rho GTPases expression. Toxicol. Appl. Pharmacol..

[53] Martucciello S., Lavric M., Toth B., Korponay-Szabo I., Nadalutti C., Myrsky E., Rauhavirta T., Esposito C., Sulic A.M., Sblattero D. (2012). RhoB is associated with the anti-angiogenic effects of celiac patient transglutaminase 2-targeted autoantibodies. J. Mol. Med..

[54] Bailly C., Waring M.J. (2019). Pharmacological effectors of GRP78 chaperone in cancers. Biochem. Pharmacol..

[55] Mujumdar N., Banerjee S., Chen Z., Sangwan V., Chugh R., Dudeja V., Yamamoto M., Vickers S.M., Saluja A.K. (2014). Triptolide activates unfolded protein response leading to chronic ER stress in pancreatic cancer cells. Am. J. Physiol. Gastrointest. Liver Physiol..

[56] Piacente S., Masullo M., De Neve N., Dewelle J., Hamed A., Kiss R., Mijatovic T. (2009). Cardenolides from *Pergularia tomentosa* display cytotoxic activity resulting from their potent inhibition of Na^+^/K^+^-ATPase. J. Nat. Prod..

[57] Hosseini S.H., Masullo M., Cerulli A., Martucciello S., Ayyari M., Pizza C., Piacente S. (2019). Antiproliferative Cardenolides from the Aerial Parts of *Pergularia tomentosa*. J. Nat. Prod..

[58] Hamed A.I., Plaza A., Balestrieri M.L., Mahalel U.A., Springuel I.V., Oleszek W., Pizza C., Piacente S. (2006). Cardenolide glycosides from *Pergularia tomentosa* and their proapoptotic activity in Kaposi’s sarcoma cells. J. Nat. Prod..

[59] Shen S., Zhang Y., Wang Z., Gong X., Liu R. (2014). Bufalin induces the interplay between apoptosis and autophagy in glioma cells through endoplasmic reticulum stress. Int. J. Biol. Sci..

[60] Martucciello S., Paolella G., Muzashvili T., Skhirtladze A., Pizza C., Caputo I., Piacente S. (2018). Steroids from *Helleborus caucasicus* reduce cancer cell viability inducing apoptosis and GRP78 down-regulation. Chem. Biol. Interact..

[61] Li J., Xia X., Ke Y., Nie H., Smith M.A., Zhu X. (2007). Trichosanthin induced apoptosis in HL-60 cells via mitochondrial and endoplasmic reticulum stress signaling pathways. Biochim. Biophys. Acta.

